# Is Floppy Eyelid Syndrome More Prevalent in Obstructive Sleep Apnea Syndrome Patients?

**DOI:** 10.1155/2016/6980281

**Published:** 2016-06-05

**Authors:** Ping Wang, Dao-Jiang Yu, Gang Feng, Zhen-Hai Long, Chang-Jiang Liu, Hui Li, Tian-Lan Zhao

**Affiliations:** ^1^Department of Plastic Surgery, The Second Affiliated Hospital of Soochow University, 1055 Sanxiang Street, Suzhou, Jiangsu 215004, China; ^2^Department of Otolaryngology Surgery, Central Hospital of Fuxin, 74 Zhonghua Street, Fuxin, Liaoning 123000, China; ^3^First Affiliated Hospital of Liaoning Medical University, Jinzhou, Liaoning 121000, China; ^4^Department of Cerebral-Neuro Surgery, Central Hospital of Fuxin, 74 Zhonghua Street, Fuxin, Liaoning 123000, China; ^5^Department of Ophthalmology, Shanghai Jiaotong University Affiliated Sixth People's Hospital, Shanghai 200233, China

## Abstract

Controversial findings are reported about the relationship between floppy eyelid syndrome (FES) and obstructive sleep apnea syndrome (OSAS). The main goal of this study was to evaluate whether FES is more prevalent in OSAS patients by performing a meta-analysis. A comprehensive literature search of Pubmed, Embase, and Cochrane databases was performed. Only studies related to the prevalence of FES in OSAS were included in the meta-analysis. We estimated a pooled odds ratio (OR) for the prevalence of FES in OSAS. In total, 6 studies with 767 participants met the inclusion criteria. Using a fixed-effects model, the pooled OR was 4.12. The test for the overall effect revealed that FES was statistically prevalent in OSAS patients when compared with that in non-OSAS subjects (*Z* = 4.98, *p* < 0.00001). In the subgroup analysis by OSAS severity, the incidence of FES in OSAS increased with severity of OSAS as indicated with increased OR values (OR = 2.56, 4.62, and 7.64 for mild, moderate, and severe OSAS). In conclusion, the results indicate that FES is more prevalent in OSAS patients. However, this result was based only on unadjusted estimates. Prospective cohort studies are needed to determine whether OSAS is an independent risk factor for FES.

## 1. Introduction

Floppy eyelid syndrome (FES) was first described by Culbertson and Ostler in 1981 [1]. It is a frequently forgotten and underdiagnosed disorder of unknown pathogenesis. FES is characterized by very elastic upper lids that became easily distorted and evertible with minimal lateral traction and chronic papillary conjunctivitis of the upper palpebral conjunctiva and typically affects obese middle-aged men [1]. The prevalence of FES within the general population varies within a range of 2.3% and 3.8% [2]. Since that first report, a growing number of publications have reported a high prevalence of other ophthalmic pathologies in patients with FES; these include corneal abnormalities [3, 4], eyelid abnormalities [5–7], and glaucoma [8]. FES has also been associated with a variety of systemic diseases such as obesity, hypertension, ischemic heart disease, diabetes mellitus, skin pathologies, and most commonly obstructive sleep apnea syndrome (OSAS) [9–11].

OSAS is characterized by recurrent episodes of partial or complete upper airway obstruction causing cessation of breathing during sleep [12]. It causes sleep disorder, waking the patient during night and resulting in excessive daytime somnolence. OSAS has high associated morbidity and mortality and is linked to hypertension, heart failure, stroke, and motor vehicle accidents [13, 14], and some cases can result in death.

Woog et al. firstly reported a possible association between FES and OSAS in 1984 in 3 patients [15]. This proposed link was further reinforced by McNab [16], in which 26 of 27 FES patients were found to have OSAS [8]. Up to now, the prevalence of FES in the OSAS population varies from 2% [17, 18] to 32% [9]. Some studies reported that there is close relationship between FES and OSAS. These studies are based on the findings that both FES and OSAS have similar pathologic changes in these two diseases, that is, a common defect in elastic tissue, and similar patient profiles, that is, middle-aged and obese men. Netland et al. were the first to find an increase in the elastolytic metalloproteinase enzymes, reporting a marked reduction in the amount of elastin fibers in the tarsal plates of patients with FES [19]. Schlötzer-Schrehardt et al. [20] demonstrated a substantial loss of elastic fibers and ultrastructural abnormalities in residual fibers, together with an increased expression of elastin-degrading enzymes in the tarsus and skin of eyelid specimens from FES patients. Other studies showed elastin fiber network disorganization and a loss of elastic fibers in the distal uvula tissue from patients with OSAS undergoing uvulopharyngoplasty [17, 19, 21]. These changes in elastic fibers provide evidence to those who have proposed a link between FES and OSAS and these changes may explain how OSA and FES could be different manifestations of the same disease.

In another way, both FES and OSAS symptoms seem to be associated with sleep posture. McNab [8] claimed that an association may exist between sleeping posture and pressure on the eye. The upper lid in FES patients has been observed to be everted during sleep and on waking. Many patients have strictly unilateral disease, and the affected side is commonly more symptomatic on the side that the patient is used to sleeping on [16, 22]. Symptoms and signs have reduced when the side the patient sleeps on has changed [4]. Recently, FES was suggested as a useful way to identify individuals with a greater probability of having glaucoma in the OSAS population [23]. All of these seem to suggest a strong link between disturbed sleep and their eyelid changes.

Others argued that both FES and OSAS are independently associated with obesity, male gender, and increasing age, which raises concern that these per se may be confounding factors. Thus it is unclear whether OSAS and FES are causally associated, whether they merely share common risk factors, or whether they have a common pathophysiological cause.

Why patients with OSAS are at risk for FES is not known. Previous results suggest that the risk is determined by an increased body mass index and the patients with FES are younger and more obese and have higher apneas or hypopneas index (AHI) than typical patients with OSAS [16, 22]. However that relationship between the presence of FES and OSAS exists even when controlling for this variable. The contradictory reports suggesting this association are less conclusive due to the small number of patients [9, 22, 24, 25], inclusion of continuous positive airway pressure-treated patients [2, 9], or lack of systematic objective assessment of sleep apnea syndrome [2, 24].

In view of the association between FES and OSAS may provide awareness for the otolaryngologist to facilitate further management. Therefore, it is necessary to make clear whether FES is more prevalent in OSAS patients. Thus, we performed this meta-analysis to evaluate the prevalence of FES in OSAS patients compared with that in non-OSAS subjects.

## 2. Materials and Methods

Our meta-analysis was conducted in strict accordance with the PRISMA (Preferred Reporting Items for Systematic Reviews and Meta-Analyses) statement, which is reporting guideline for meta-analyses [26]. The search was performed in the Pubmed, Embase, and Cochrane databases up to December 2015. The search terms were “floppy eyelid” OR “floppy eyelid syndrome” AND “obstructive sleep apnea” OR “obstructive sleep apnea syndrome” OR “obstructive sleep apnea hypopnea syndrome” OR “OSA” OR “OSAS” OR “OSAHS”. The language of publication was limited within English. Additionally, we manually searched for relevant published studies and review articles.

The inclusion criteria for the current meta-analysis were the following: (1) only studies that were of an observational design and concerned the prevalence of FES in OSAS patients; (2) only studies that obtained consent from the patients; (3) only studies where polysomnography or oximetry was used and definition of hypopnea and apnea was presented for diagnosis of OSAS; (4) only studies where FES and OSAS could be used as an outcome in the analysis; and (5) only studies with an effective control group.

Two investigators (Dr. Ping Wang and Dao-Jiang Yu) independently screened all identified studies using the above-mentioned criteria. When any disagreement emerged, a third reviewer (Dr. Tian-Lan Zhao) participated in the resolution of the issue by discussion.

### 2.1. Meta-Analysis

Meta-analyses were then conducted regarding the prevalence of FES in OSAS by calculating odd ratio (OR) with 95% confidence interval (CI). Cochrane's *I* ^2^ index was calculated to assess heterogeneity, and if the data were not significant (*p* > 0.05, *I* ^2^ < 40%), the ORs were pooled according to the fixed-effect model. Otherwise, the random-effect model was used. The statistical significances of the pooled ORs were evaluated using the *Z*-test. Stratified analysis was performed by severity of OSAS. Possible publication bias was assessed with funnel plots. The meta-analyses were performed using the Review Manager (RevMan, version 5.2) from the Cochrane Collaboration [27, 28].

### 2.2. Quality Assessment

Because there is no consensus as to the “best” standardized method for assessing the quality of observation studies [29], we designed a five-item scoring scale (each item scoring 0 or 1; 1 being better) [29, 30]. The items on the integer scale were representativeness of the cases, whether the diagnosis criteria of FES were given, whether the assessment of OSAS was objective, and whether OSAS severity was assessed and controls for confounding factors. Score of 0–3 was evaluated as “low” quality while 4 or 5 was considered to indicate “high” quality [29, 30]. The quality of each study was independently assessed by two investigators (Dr. Hui Li and Gang Feng). The quality scores of the included studies are shown in Table 1.

## 3. Results

### 3.1. Search Results

**Figure 1:**
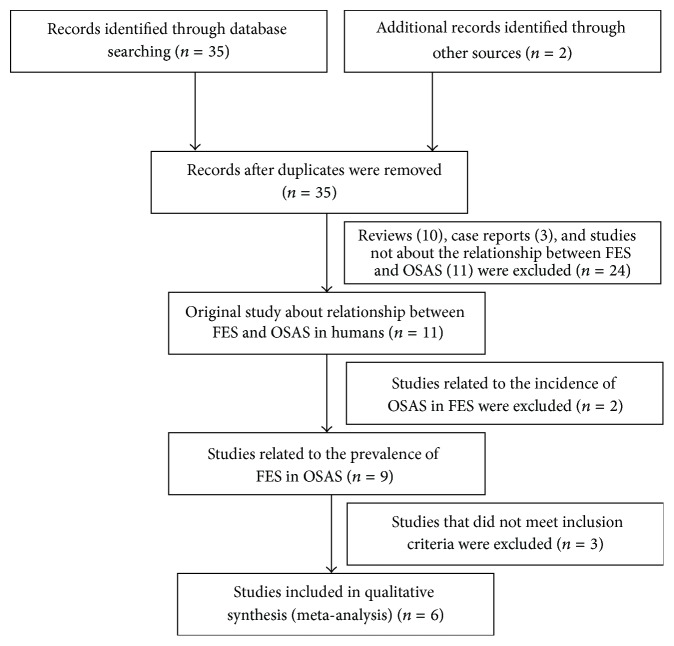
Flow diagram of the study identification, eligibility, and inclusion process.

The articles were initially identified by electronic and manual searching. After a review of the titles and abstracts, we excluded reviews, case reports, letters, and studies not about the relationship between FES and OSAS. The resultant eleven studies are related to the relationship between FES and OSAS. As the aim of this meta-analysis is to review whether FES is more prevalent in OSAS, two articles addressing the prevalence of OSAS in FES were excluded. Among the nine studies about the prevalence of FES in OSAS, three studies that did not meet the inclusion criteria were excluded. Finally six studies that met the inclusion criteria were included in the meta-analysis. The flow chart of the article selection process is shown in Figure 1.

### 3.2. Characteristics of Included Studies

First, the diagnosis criteria for OSAS were somewhat different among all the included studies. Most studies adopted AHI to diagnose OSAS [18, 31–33], while others used oxygen desaturation index [2] or respiratory disturbance index [9]. Second, the definitions for AHI and the grouping criteria for OSAS were slightly different in studies with AHI as diagnosis criteria. All these may affect the prevalence of FES in OSAS, especially that in different OSAS severity group. Third, the diagnosis criteria for FES were subjective in all included studies [2, 18, 31–33] except for one study without any diagnosis criteria [9]. Finally, the relationship between FES and OSAS is based on the hypothesis of the study. For example, the expression of FES and OSAS which could be different manifestations of the same disease was referred to in two studies and the prevalence of FES in OSAS patients was higher than non-OSAS population [31, 32].

### 3.3. Pooled-Analysis Results

The meta-analysis data were derived from 6 studies of 609 cases in the OSAS group and 158 cases in the control group [2, 9, 18, 31–33].

**Figure 2:**
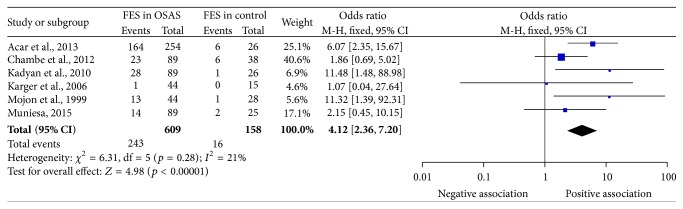
Forest plot of FES prevalence in OSAS. FES, floppy eyelid syndrome; OSAS, obstructive sleep apnea syndrome; CI, confidence interval.

As shown in Figure 2, the heterogeneity was not statistically significant (*I* ^2^ = 21%, *p* = 0.28), and thus a fixed-effect model was used. The pooled OR and 95% CI were 4.12 and 2.36~7.20, respectively. The test for the overall effect revealed that FES was statistically prevalent in OSAS patients when compared with that in non-OSAS subjects (*Z* = 4.98, *p* < 0.00001).

In the subgroup analysis by OSAS severity, the heterogeneity was not statistically significant (*I* ^2^ = 0%, *p* = 0.62; *I* ^2^ = 21%, *p* = 0.28; and *I* ^2^ = 13%, *p* = 0.32 for mild, moderate, and severe subgroup, resp.), and thus fixed-effect models were used in the following analysis.

**Figure 3:**
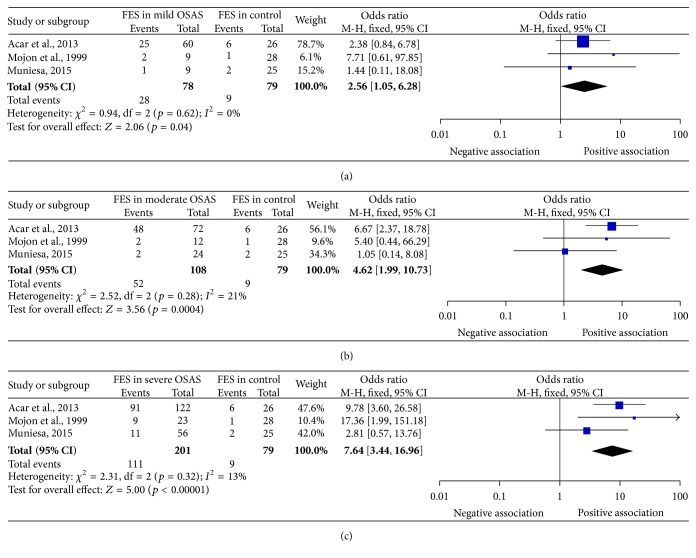
Forest plot of FES prevalence in OSAS subgroup according to severity of the disease. (a) FES prevalence in mild OSAS; (b) FES prevalence in moderate OSAS; (c) FES prevalence in severe OSAS. FES, floppy eyelid syndrome; OSAS, obstructive sleep apnea syndrome; CI, confidence interval.

The test for the overall effect revealed FES was statistically prevalent in OSAS patients when compared to that in non-OSAS subjects, regardless of whether the severity is mild (*p* value of *Z*-test = 0.04), moderate (*p* value of *Z*-test = 0.0004), or severe (*p* value of *Z*-test < 0.0001). The incidence of FES in OSAS increased with severity of OSAS as indicated with increased OR values (OR = 2.56, 4.62, and 7.64 for mild, moderate, and severe subgroups, resp.). The 95% CI for mild, moderate, and severe subgroups were 1.05–6.28, 1.99–10.73, and 3.44–16.96, respectively. The subgroups analysis results were summarized in Figure 3 and Table 2.

### 3.4. Sensitivity Analysis

To evaluate the sensitivity of the meta-analysis, each study was sequentially excluded from the meta-analysis, and the corresponding heterogeneity results and results of the tests for overall effect are shown in Table 3. As shown in Table 3, the heterogeneities and overall effect of prevalence of FES in OSAS did not alter significantly when excluding any study from the meta-analysis, with the heterogeneity changing between 0% and 30% and all *p* values of overall effect remaining less than 0.0005.

### 3.5. Publication Bias Analysis

**Figure 4:**
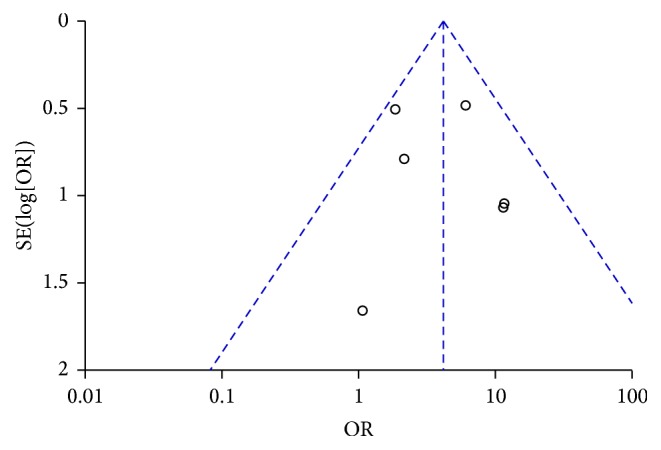
Funnel plot of the prevalence of FES in OSAS. FES, floppy eyelid syndrome; OSAS, obstructive sleep apnea syndrome.

Funnel plot was performed to assess the publication bias of literatures. The shape of the funnel plot was asymmetric (Figure 4) which suggested that the publication bias may exist.

## 4. Discussion

In this meta-analysis, we investigated the prevalence of FES in OSAS including 767 subjects. We found that individuals with OSAS showed an increased risk of FES in the overall population. The result from our meta-analysis suggested that OSAS patients had 4.12 times higher FES risk compared to those non-OSAS individuals.

Among the six included studies in this meta-analysis, four studies suggested that FES is more prevalent in OSAS patients and two studies did not report the close relationship between FES and OSAS [18, 33]. The main result of meta-analysis is consistent with most of the included studies. Actually the prevalence of FES in OSAS is greatly different among all the included studies, varying from 2.27% (1/44) [18] to 64.57% (164/254) [32]. This difference may arise from the different definitions for apnea or hypopnea and different criteria for OSAS severity grouping in these studies. As demonstrated in Table 1, three studies used the American Academy of Sleep Medicine 2007 criteria in sleep staging and in determining the respiratory pathologies. The hypopnea was defined as a 30% reduction in airflow accompanied by a 4% oxygen desaturation or a 50% reduction in airflow accompanied by a 3% oxygen desaturation or arousal [9, 31, 32], while in Karger et al.'s study [18] obstructive apnea was defined as cessation of airflow despite respiratory effort for at least 10 s and hypopnea was defined as at least a 30% drop in airflow for at least 10 s despite respiratory effort and at least a 4% drop in oxyhemoglobin saturation. In the study of Muniesa Royo et al. [33], obstructive apnea was defined as an absence of airflow for at least 10 s and hypopnea was defined as a clear (50%) airflow reduction for at least 10 s, with a drop in oxygen saturation of at least 4% or an arousal. The differences are whether or not to include the time of airflow reduction and the amount of airflow reduction when determining a respiratory event. The definition of respiratory event is the basic for diagnosis of OSAS and thus may impact the grouping of OSAS and non-OSAS population and severity of OSAS. In addition, one study [2] used only oximetry, which is a method not considered valid for OSAS diagnosis [23]. All these detailed differences may impact the prevalence of FES in OSAS. In diagnosis of FES, subjectively easy eyelid eversion is the current gold standard in assessing eyelid laxity. This may be based on the experience of the examiner and thus impact the occurrence rate of FES in OSAS patients.

In the stratified analysis by OSAS severity, the significant prevalence was observed in mild, moderate, and severe OSAS and the FES risk increased with the severity of OSAS with OR as 2.56, 4.62, and 7.64 from mild to severe OSAS. This is consistent with previous report showing that a floppy eyelid rate was positively correlated with respiratory disturbance index values [9, 32]. As AHI values increase, the clinical course of the OSAS becomes more severe, the tissue mechanic stress increases, and the eyelid floppiness progresses due to hypoxemia. However, only three studies reported FES prevalence in different severity of OSAS [9, 32, 33] and the sample number was small. Thus, the results lacked sufficient reliability to confirm or refute an association in a definitive manner. In the future, more studies with FES in different severity of OSAS are still needed to address the role of OSAS on FES risk.

The higher prevalence of FES in OSAS patients may indicate that the improvement of accompanying systemic and ocular symptoms may be expected after the appropriate treatment of OSAS. This was confirmed by a study in which the symptoms and signs of FES disappeared after continuous positive airways pressure during sleep [4]. The author indicated the change of mechanical force to the eyelid during sleep, which varied with body position from prone to supine as the continuous positive airways pressure treatment progresses, may help alleviate the symptoms of FES. Specifically, patients with OSAS may initially sleep prone; the mechanical pressure to the eyelid is bigger. As continuous positive airways pressure treatment progresses, the symptoms of OSAS may be relieved. Consequently they may lay supine; the mechanical pressure to the eyelid diminishes. Finally the symptoms of FES disappeared. Certainly, this hypothesis needs to be confirmed with large number of samples in the future. If this is the case, it may also provide another therapy choice for FES patients and ophthalmologists, although the reversal of FES with continuous positive airways pressure in OSAS patients was not common. Given the strength of the association between FES and OSAS, the current findings raise the questions of whether FES screening should be performed routinely in OSAS patients. Sleep physicians and ophthalmologists should be aware of this association and thus refer suspected patients for diagnosis and therapy.

The limitation of this meta-analysis is that there is a referral bias in the population studied, as none of the studies selected their participants randomly. Most were based on patients attending a clinic for evaluation of OSAS and without therapy except one study including OSAS patients after continuous positive airway pressure therapy [18]. This may not be representative of the entire OSAS population, majority of whom remain undiagnosed; thus selection bias may exist. The referred group of patients would include those with significant symptoms and identified as suspects by the general practitioner in the community. Meanwhile, the control group in these included studies, which constitutes suspects of OSAS and is referred to sleep center, raises the question of whether the non-OSAS patients are representative of the general population. It is expected that this non-OSAS had higher AHI than general population because they all had symptoms of OSAS more or less. From this point of view, the occurrence rate difference between FES in OSAS and FES in the true sense of non-OSAS population should be more statistically significant. This indicated that the pooled results in this study represent unadjusted estimates, which are likely to be confounded by other risk factors; these results should be confirmed in further studies. Thus it is difficult to draw a definitive conclusion from this meta-analysis because of the different quality assessment of the included studies.

In conclusion, this study confirmed that FES is very common in OSAS but that FES is not so common among the general non-OSAS population. Large prospective randomized studies are required to better elucidate the exact prevalence of FES in patients with OSAS and the direct prognostic value.

## Figures and Tables

**Table 1:** Characteristics of the studies included in meta-analysis.

Study	Mean age (years), non-OSAS/OSAS	Sex (F/M)	OSAS diagnosis	FES diagnosis	FES in OSAS	FES in non-OSAS	Statistically significant	Study quality score
Mojon et al., 1999 [9]	Non-OSAS: 48.7 ± 13.7; mild: 51.1 ± 9.2; moderate: 56.0 ± 11.1; severe: 53.6 ± 8.1	Non-OSAS: 6/22; mild: 0/9; moderate: 2/10; severe: 4/19	PSG. Hypopnea was defined as a 30% reduction in airflow accompanied by a 4% oxygen desaturation or a 50% reduction in airflow accompanied by a 3% oxygen desaturation or arousal. Non-OSAS: RDI < 10; mild: 10 ≤ RDI < 20; moderate: 20 ≤ RDI < 40; severe: AHI ≥ 40	Not presented	Mild: 2/9; moderate: 2/12; severe: 9/23; overall: 13/44	1/28	Yes	3

Karger et al., 2006 [18]	Non-OSAS: 43.2 ± 11.82; OSAS: 62.7 ± 15.02	Non-OSAS: 7/8; OSAS: 16/28	PSG. Obstructive apnea was defined as cessation of airflow despite respiratory effort for at least 10 s. Hypopnea was defined as at least a 30% drop in airflow for at least 10 s despite respiratory effort and at least a 4% drop in oxyhemoglobin saturation	Subjectively easy eversion, papillary conjunctivitis, and lash ptosis	1/44	0/15	No	4

Kadyan et al., 2010 [2]	Non-OSAS: 55.3 ± 10.7; OSAS: 55.75 ± 10.97	Non-OSAS: 9/17; OSAS: 14/75	Oximetry. ODI: events of 4% oxygen desaturation rate per hour. Non-OSAS: ODI < 5; mild: 5 ≤ ODI < 15; moderate: 15 ≤ ODI < 30; severe: ODI > 30	Subjectively easy eversion, papillary conjunctivitis, and symptoms of ocular irritation	28/89	1/26	Yes	5

Chambe et al., 2012 [31]	Non-OSAS: 45.6 ± 1.9; mild: 53.8 ± 1.9; moderate: 56.8 ± 2.0; severe: 59.2 ± 1.4	Non-OSAS: 19/19; mild: 21/22; moderate: 7/19; severe: 4/19	PSG. Hypopnea was defined as a 30% reduction in airflow accompanied by a 4% oxygen desaturation or a 50% reduction in airflow accompanied by a 3% oxygen desaturation or arousal. Non-OSAS: AHI ≤ 5; mild: 5 < AHI ≤ 15; moderate: 15 < AHI ≤ 30; severe: 30 < AHI	Eyelid hyperlaxity and papillary conjunctivitis	23/89	6/38	Yes	5

Acar et al., 2013 [32]	Non-OSAS: 46.7 ± 9; mild: 43.9 ± 11.5; moderate: 49.9 ± 9.3; severe: 48.1 ± 10.5	Non-OSAS: 16/10; mild: 17/43; moderate: 28/44; severe: 22/100	PSG. Hypopnea was defined as a 30% reduction in airflow accompanied by a 4% oxygen desaturation or a 50% reduction in airflow accompanied by a 3% oxygen desaturation or arousal. Non-OSAS: AHI < 5; mild: 5 ≤ AHI < 15; moderate: 15 ≤ AHI ≤ 30; severe: 30 < AHI	Subjectively easy eversion and tarsal conjunctiva	Mild: 25/60; moderate: 48/72; severe: 91/122; overall: 164/254	6/26	Yes	4

Muniesa Royo et al., 2013 [33]	Non-OSAS: 48.6 ± 11.15; OSAS: 55.1 ± 9.41	Non-OSAS: 7/18; OSAS: 21/67	PSG or cardiorespiratory sleep study. Obstructive apnea was defined as an absence of airflow for at least 10 s and hypopnea was defined as a clear (50%) airflow reduction for at least 10 s, with a drop in oxygen saturation of at least 4% or an arousal. Non-OSAS: AHI < 10; mild: 10 ≤ AHI < 20; moderate: 20 ≤ AHI ≤ 30; severe: AHI > 30	Subjectively easy eversion and papillary conjunctivitis	Mild: 1/9; moderate: 2/24; severe: 11/56; overall: 14/89	2/25	No	5

OSAS: obstructive sleep apnea/hypopnea syndrome; PSG: polysomnography; RDI: respiratory disturbance index; AHI: apneas or hypopneas index; ODI: oxygen desaturation index; BMI: body mass index.

**Table 2:** Results of subgroup analysis. FE: fixed effect.

Severity of OSAS	Number of studies	Weight of the studies (%)			Test of overall effect			Model	Heterogeneity		
		Mojon et al., 1999 [9]	Acar et al., 2013 [32]	Muniesa Royo et al., 2013 [33]	OR (95% CI)	*Z*	*p* value		*χ* ^2^	*p* value	*I* ^2^ (%)
Mild	3	6.1	78.7	15.2	2.56 (1.05, 6.28)	2.06	0.04	FE	0.94	0.62	0
Moderate	3	9.6	56.1	34.3	4.62 (1.99, 10.73)	3.56	0.0004	FE	2.52	0.28	21
Severe	3	10.4	47.6	42.0	7.64 (3.44, 16.96)	5.00	<0.0001	FE	2.31	0.32	13

**Table 3:** Results of the sensitivity analysis.

Excluded study	FES in OSAS				
	Heterogeneity			Overall effect	
	*χ* ^2^	*I* ^2^	*p* value	*Z* value	*p* value
Mojon et al., 1999 [9]	5.10	22%	0.28	4.41	<0.0001
Karger et al., 2006 [18]	5.71	30%	0.22	5.02	<0.0001
Kadyan et al., 2010 [2]	4.97	20%	0.29	4.28	<0.0001
Chambe et al., 2012 [31]	3.41	0%	0.49	4.92	<0.00001
Acar et al., 2013 [32]	4.92	19%	0.30	3.59	=0.0003
Muniesa Royo et al., 2013 [33]	5.74	30%	0.22	4.96	<0.00001
